# Supplementary material for: Hydrophobic Homopolymer’s Coil–Globule Transition and Adsorption onto a Hydrophobic Surface under Different Conditions
Source: J Phys Chem B. 2023 Jun 19;127(25):5541–52. doi: 10.1021/acs.jpcb.3c00937 (PMC10316403; doi:10.1021/acs.jpcb.3c00937)
Supplement: Supplementary file 1 — jp3c00937_si_001.zip [file jp3c00937_si_001.zip › Suppl-DuraBiancoFranzese-v3.pdf]

Hydrophobic homopolymer's coil-globule transition and adsorption onto a hydrophobic surface under different conditions

Bernat Durà Faulí,† Valentino Bianco,‡ and Giancarlo Franzese*,†,¶

†*Secció de Física Estadística i Interdisciplinària - Departament de Física de la Matèria Condensada, Universitat de Barcelona, Martí i Franquès 1, 08028 Barcelona, Spain.*

‡*Onena Medicines S.L., Paseo Miramón, 170, planta 3, B06, 20014, Donostia, Gipuzkoa, Spain.*

¶*Institut de Nanociència i Nanotecnologia, Universitat de Barcelona, 08028 Barcelona, Spain.*

E-mail: gfranzese@ub.edu

May 9, 2023

Supporting Information

Table S1: Model's parameters with or without top-down symmetry. Those not indicated in the table are the same in both cases and are: $v_{\mathrm{HB}}/v_0 = 0.5$, $J/4\epsilon = 0.3$, $J_\sigma/4\epsilon = 0.05$, $k_1 = v_0/4\epsilon$, with units $r_0 \equiv v_0^{1/3} = 2.9$ Å, $\epsilon = 5.8$ kJ/mol.

**Model's parameter**	$\Delta J^{(\phi)}/J$
without top-down symmetry	0.83
with top-down symmetry	0.17

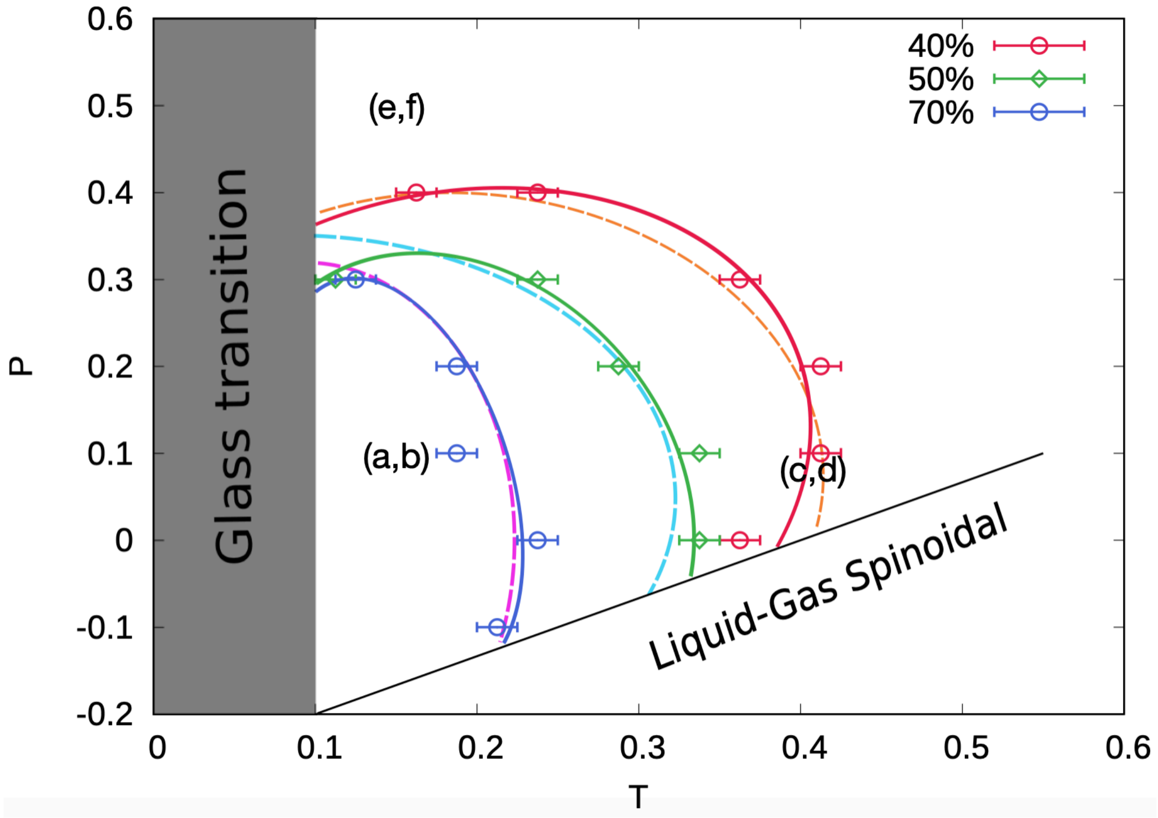

Figure S1: As in Fig. 4 but without the bias. Lines are guides for the eyes. The change in liquid-gas spinodal is the same with or without the bias. On the other hand, the change in SR without bias is negligible. Lines and symbols are as in Fig. 4. Because we reduce $\Delta J^{(\phi)}/J$ in the case without bias (Table S1), the SR is less accessible compared to Fig. 4. The labels (a,b), (c,d), etc., refer to the state points discussed in Fig. S2.

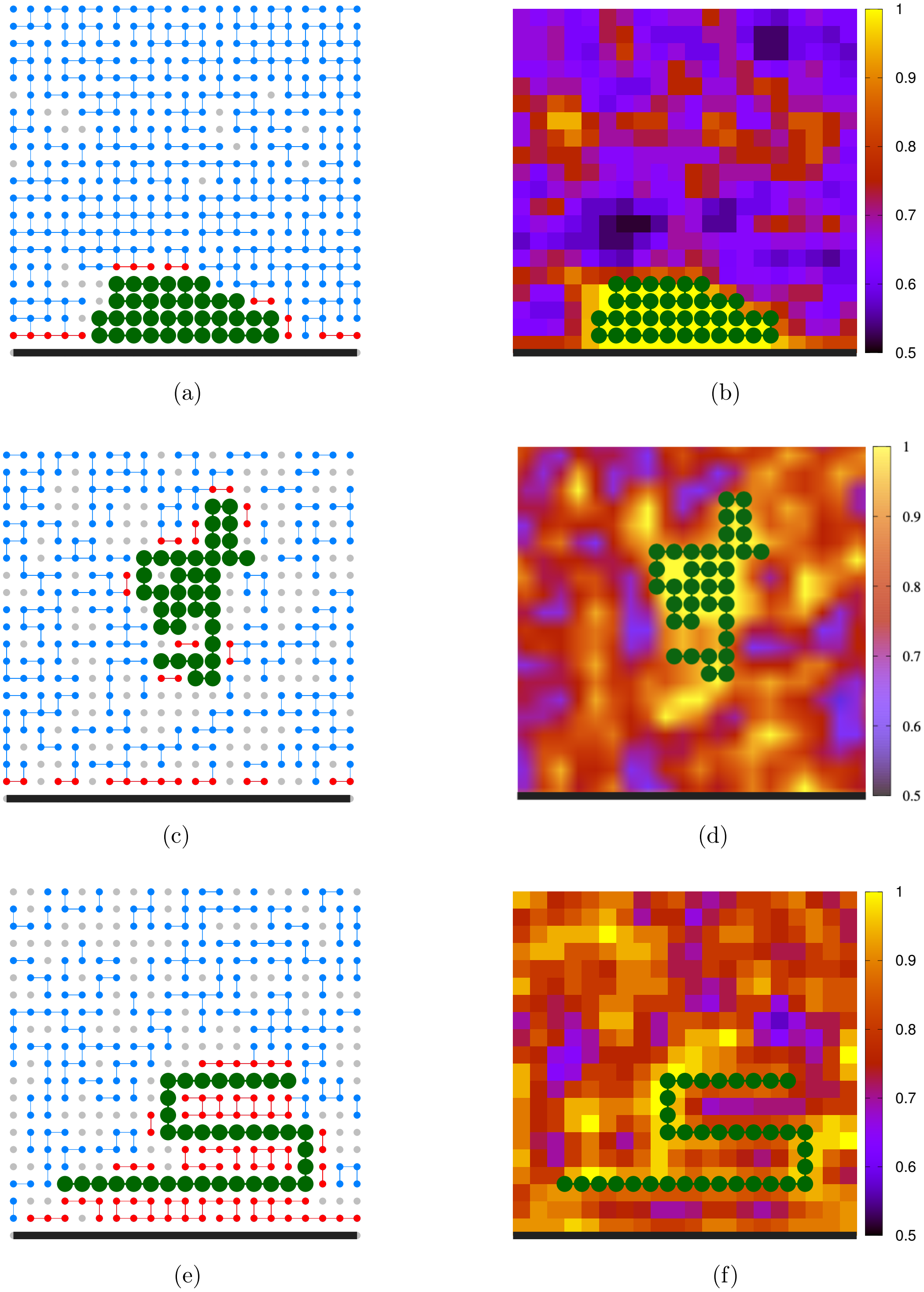

Figure S2: As in Fig. 5 but without the bias. The thermodynamic state points $(k_BT/4\epsilon, Pv_0/4\epsilon)$ of the panels are reported in the phase diagram in Fig. S1: **(a), (b)** (0.15, 0.10); **(c), (d)** (0.40, 0.10); **(e), (f)** (0.15, 0.50).

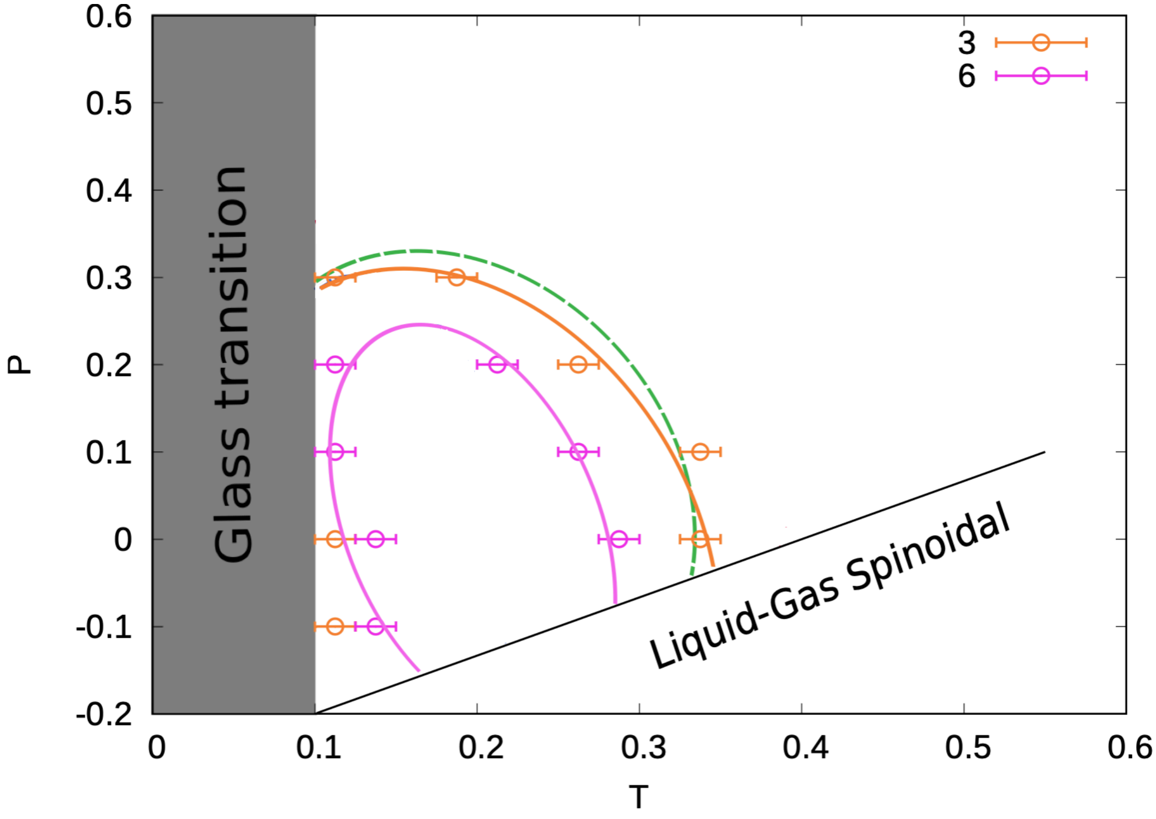

Figure S3: Regions where the homopolymer-interface average number of contact points is > 3 (orange symbols and line) and > 6 (indigo symbols and line) compared to the SR at 50% collapse (dashed green line from Fig. S1) for the confined case without bias. Lines are guides for the eyes. The polypeptide adsorbs when it collapses in the unbiased case. However, at T near the glassy state and $P \leq 0$, the extremely slow chain diffusion can prevent it from reaching the interface and adsorbing onto it.
